# Cost-effectiveness of reducing children’s sedentary time and increasing physical activity at school: the Transform-Us! intervention

**DOI:** 10.1186/s12966-024-01560-3

**Published:** 2024-02-12

**Authors:** Vicki Brown, Lauren Sheppard, Jo Salmon, Lauren Arundell, Ester Cerin, Nicola D. Ridgers, Kylie D. Hesketh, Robin M. Daly, David W. Dunstan, Helen Brown, Jacqueline Della Gatta, J. M. M. Chinapaw, Marj Moodie

**Affiliations:** 1 Deakin University, Deakin Health Economics, Global Centre for Preventive Health and Nutrition (GLOBE), Institute for Health Transformation (IHT), Geelong, Australia; 2 Deakin University, Deakin Health Economics, Institute for Health Transformation, Geelong, Australia; 3 Deakin University, Institute for Physical Activity and Nutrition (IPAN), Geelong, Australia; 4 Mary McKillop Institute for Health Research, Australian Catholic University, Melbourne, Australia; 5 Allied Health and Human Performance, Alliance for Research in Exercise, Nutrition and Activity (ARENA), University of South Australia; Deakin University, Institute for Physical Activity and Nutrition, Geelong, Australia; 6 Baker Heart and Diabetes Institute, Melbourne, Australia; 7 Department of Public and Occupational Health, Amsterdam UMC, Vrije Universiteit Amsterdam, Amsterdam Public Health research institute, Amsterdam, Netherlands

**Keywords:** Economic evaluation, Cost-effectiveness, Physical activity, Sedentary behavior

## Abstract

**Background:**

Improving physical activity and reducing sedentary behavior represent important areas for intervention in childhood in order to reduce the burden of chronic disease related to obesity and physical inactivity in later life. This paper aims to determine the cost-effectiveness of a multi-arm primary school-based intervention to increase physical activity and/or reduce sedentary time in 8–9 year old children (*Transform-Us!*).

**Methods:**

Modelled cost-utility analysis, using costs and effects from a cluster randomized controlled trial of a 30-month intervention that used pedagogical and environmental strategies to reduce and break up sedentary behaviour (SB-I), promote physical activity (PA-I), or a combined approach (PA + SB-I), compared to current practice. A validated multiple-cohort lifetable model (ACE-Obesity Policy model) estimated the obesity and physical activity-related health outcomes (measured as change in body mass index and change in metabolic equivalent task minutes respectively) and healthcare cost-savings over the cohort’s lifetime from the public-payer perspective, assuming the intervention was delivered to all 8–9 year old children attending Australian Government primary schools. Sensitivity analyses tested the impact on cost-effectiveness of varying key input parameters, including maintenance of intervention effect assumptions.

**Results:**

Cost-effectiveness results demonstrated that, when compared to control schools, the PA-I and SB-I intervention arms were “dominant”, meaning that they resulted in net health benefits and healthcare cost-savings if the intervention effects were maintained. When the costs and effects of these intervention arms were extrapolated to the Australian population, results suggested significant potential as obesity prevention measures (PA-I: 60,780 HALYs saved (95% UI 15,007-109,413), healthcare cost-savings AUD641M (95% UI AUD165M-$1.1B); SB-I: 61,126 HALYs saved (95% UI 11,770 − 111,249), healthcare cost-savings AUD654M (95% UI AUD126M-1.2B)). The PA-I and SB-I interventions remained cost-effective in sensitivity analysis, assuming the full decay of intervention effect after 10 years.

**Conclusions:**

The PA-I and SB-I *Transform-Us!* intervention arms represent good value for money and could lead to health benefits and healthcare cost-savings arising from the prevention of chronic disease in later life if intervention effects are sustained.

**Trial registration:**

International Standard Randomized Controlled Trial Number (ISRCTN83725066). Australia and New Zealand Clinical Trials Registry Number (ACTRN12609000715279).

**Supplementary Information:**

The online version contains supplementary material available at 10.1186/s12966-024-01560-3.

## Background

Childhood obesity, physical inactivity and sedentary behaviours, such as sitting, are prevalent globally [1]. Overweight and obesity can lead to increased risk of health conditions in both childhood and adulthood [2] and the health benefits of physical activity are widely acknowledged [3]. While the role of prolonged sedentary behaviours on health outcomes is currently less clear, replacing sedentary time with physical activity could have beneficial health effects [4]. Since the lifetime economic burden of overweight and obesity in childhood is high [5], interventions to encourage physical activity and healthy body weight and reduce sedentary behaviours among children are warranted.

Children spend a large proportion of their waking hours at school, which represents an accessible intervention setting irrespective of each child’s background characteristics [6]. School-based interventions targeting physical activity and sedentary behaviours have been seen as promising in reducing or preventing obesity [7]. While the effectiveness of school-based interventions has been examined in several systematic reviews, the results have been mixed [6, 8, 9]. Studies suggest that a large degree of heterogeneity exists in the frequency, duration and content of school-based interventions, and that more focus is needed on understanding both the equity effects and the implementation processes of these complex interventions [6, 8, 9]. Given the multitude of competing demands for both health and education resources, it is not only important that these interventions are effective, but they must also represent “good value for money”. To date, limited economic evidence for school-based interventions targeting physical activity or sedentary behaviours has been published, with a recent systematic review of school-based lifestyle interventions identifying 23 studies in total (18 of which included physical activity-related intervention components) [10]. The authors noted that heterogeneity in the measurement, valuation and extrapolation of costs and outcomes limited comparability of findings [10]. This limited comparability may impact the usefulness of economic evaluations of school-based interventions in informing the allocation of public budgets [10]. 

The *Transform-Us!* study aimed to determine the effects of an intervention delivered in the school and home settings on children’s physical activity and sedentary behaviours, as well as cardiometabolic risk factors, at 18- and 30-months compared to current practice [11]. Methods and short-term effects for the cluster randomised controlled trial (cRCT) conducted from 2010 to 2012 have been published elsewhere [11–13], and main outcome results have been published [14]. This paper presents the cost-effectiveness results, providing evidence of the economic credentials of investment in *Transform-Us!* as an obesity prevention intervention in 8–9 year old children.

## Methods

The recommendations of the Second Panel on Cost-Effectiveness and the Consolidated Health Economic Evaluation Reporting Standards (Additional File 1) guided the analysis where practicable [15, 16]. Costs and outcomes were estimated by a comparison of the intervention and control arms, using intention-to-treat principles within the trial sample (*n* = 1,606 children). Modelled cost-effectiveness analysis estimated incremental cost-effectiveness ratios, incorporating lifetime health and cost outcomes attributable to the intervention within the trial sample. Trial costs and outcomes were then extrapolated to the Australian population of Year 3 students in Government schools (approximately 69% of all fulltime Year 3 children in 2010 (corresponding with the first year of the cRCT), *n* = 184,547 children aged approximately 8–9 years old) [17], to estimate the potential lifetime health and cost outcomes attributable to the intervention should it be delivered comprehensively throughout Australia. Modelled cost-effectiveness was estimated using the well-validated ACE-Obesity Policy model [18–22]. 

### The intervention

The *Transform-Us!* trial was a four-arm cRCT with a 2 × 2 factorial design, conducted in 20 primary schools in Melbourne, Australia [11, 12]. Approval for the trial was obtained from the Deakin University Human Research Ethics Committee (EC141-2009), the Victorian Department of Education and Early Childhood Development (2009_000344) and the Victorian Catholic Education Office (1545).

In summary, 1,606 children in Year 3 (mean age 8.3 years) at baseline were randomised by school to one of four groups: SB-I targeting reductions in sedentary behaviours; PA-I targeting increases in physical activity; PA + SB-I combining PA-I and SB-I strategies; or, usual curriculum control (C). Schools in low (*n* = 74), mid (*n* = 74) and high (*n* = 71) socioeconomic status areas were randomly ordered with probabilistic weighting according to enrolment number, and invited to participate. Eight schools from low socioeconomic status areas, 11 schools from mid socioeconomic status areas and 1 school from a high socioeconomic status area agreed to participate. For randomization, schools in mid and high socioeconomic status areas were combined and schools within each of the two strata were randomly allocated using computer-generated blocks of four by a statistician not involved in the trial [11]. Each intervention group included Year 3 students across four classes in five intervention schools (20 classes per intervention group). Whilst all children in those classes received the intervention, only those with written consent from their parents participated in the evaluation of the program. The intervention comprised an intensive period with annual teacher face-to-face professional development (first 18 months), and incorporated a mixture of educational, pedagogical, behavioral, social and environmental strategies delivered in the school and, to a lesser extent, home environment [11]. The intervention was followed by 12 months of maintenance in which teachers self-managed intervention delivery. The intervention has been described elsewhere in detail [11].

SB-I teachers were provided with 18 lesson plans incorporating key learnings about the importance of reducing sedentary behaviors. Teachers focused on reducing and breaking up children’s sedentary time during lessons and each classroom received a set of standing easels to help facilitate standing. Homework tasks encouraged students to stand or move and parent newsletters suggested strategies to reduce children’s sitting time at home. PA-I teachers were provided with 18 lesson plans with key learnings about the importance of being physically active. Play equipment and a set of pedometers was also provided for each classroom. Asphalt line markings were added to school grounds to facilitate physical activity. Homework tasks and parent newsletters reinforced the class lessons. The PA + SB-I intervention combined the SB-I and PA-I interventions.

### The comparator

Consistent with previous research, current practice was assumed to be equivalent to ‘no intervention’ and accordingly incurred no cost [23]. Control schools were supplied intervention materials on study completion.

### Perspective, time horizon, discount rate

The analysis was undertaken from the public-payer perspective. This perspective was identified as most relevant to capture the costs and benefits to both the health and education sectors, given that within the Australian setting universal health and education systems exist. The time horizon for an economic evaluation is the duration over which health outcomes and costs are calculated. The choice of time horizon is important, with longer time horizons recommended for chronic conditions where the accumulation of costs and benefits over time may influence the decision problem [24]. The time horizon for modelling costs and benefits was defined as rest of life or 100 years. Discounting was applied to both costs and benefits at the commonly accepted 3% discount rate [25]. 

### Measurement of intervention costs

The major categories of resource use identified through pathway analysis were: (i) teacher time to prepare intervention delivery; (ii) implementation costs, including equipment provided to schools; and (iii) ongoing costs required if the program was rolled out in the future (e.g., production/distribution costs of newsletters). Costs associated with intervention design and development and the time contributions of children were excluded.

Intervention group teachers recorded extra time expended in preparation for delivery of an intervention activity in a diary alongside the cRCT. Diaries were completed at regular time points throughout the 18-month intervention period, providing 19 weeks of resource use data; in addition, they were completed for an additional week during the 12-month maintenance period. It was assumed that the extra preparation time for delivery of an intervention activity could not exceed the expected duration of that activity (e.g., for an expected 30 min standing lesson we assumed that the preparation time could not exceed 30 min). Where teachers recorded extra preparation time equal to, or exceeding, the expected duration of a *Transform-Us!* activity we excluded these data from the base case cost analysis as it was assumed that a teacher had misunderstood the need to record only extra preparation time. This was tested in sensitivity analysis. Teacher time to deliver the intervention activity was not included, according to opportunity cost principles, as teachers would otherwise have been required to spend this time delivering similar or other curriculum content. In calculating annual costs, it was assumed that there were 41 school weeks per year [26]. 

Unit prices for intervention equipment were sourced from trial expenses, or major online providers. A five-year life span was assumed for all school equipment and annuitized costs were calculated by applying a 3% discount rate. Published hourly rates for teachers were adjusted to the 2010 reference year [27]. Salary costs of an Administrative Officer employed by each Australian State and Territory government (*n* = 8) were included when extrapolating intervention costs and effects to the Australian population of Year 3 children, to cater for the extra complexity of coordinating the intervention at scale.

### Measurement of effectiveness

Measures of intervention effectiveness were based on detailed published study results [14]. Intervention effects (comparison between each experimental group and the control group) related to sedentary time and BMI at 30 months were assessed for statistical significance (*p*-value < 0.05) [14]. Sedentary time was measured using accelerometry (ActiGraph GT3X (Pensacola, FL). Children’s height and weight were measured by trained research staff and used to estimate BMI z-score.

There was a statistically significant intervention effect among participants in the SB-I arm for BMI z-score at 30 months (-0.14 BMI-z (95%UI -0.26:-0.03)), as well as for sedentary time (-62.8 min per weekday (95%UI -92.0:-33.9)) compared to the control group. There was a statistically significant intervention effect among participants in the PA-I arm for BMI z-score (-0.13 BMI-z (95%UI -0.24:-0.03), but no statistically significant intervention effect on sedentary time. Participants in PA + SB-I did not report a statistically significant intervention effect for sedentary time or BMI z-score. Full reporting on measurement of intervention effectiveness has been published [11, 13, 14]. 

Statistically significant reductions in sedentary time were converted to a change in metabolic equivalent task (MET) minutes per week [28]. It was assumed that the intervention resulted in the net difference in MET minutes between time spent sitting talking (MET value 1.4) and time spent standing talking (MET value 1.8) [28]. This assumption may underestimate results if the reduction in sedentary time equated to a change from sitting to an activity with a higher MET value (e.g., walking-light effort (MET value 2.9) [28]). Statistically significant reductions in sedentary time (minutes per weekday) were multiplied by the difference in MET values (0.4MET), to estimate the change in MET minutes arising from intervention.

### Measurement of cost-effectiveness

A proportional multi-state lifetable Markov cohort model (the ACE-Obesity Policy model) was used to estimate cost-effectiveness [18–22, 29]. The validated ACE-Obesity Policy model was developed as part of a large priority-setting study undertaken in Australia from 2012 to 2018 [18–22, 29]. Markov modelling is widely used in economic evaluation, and is particularly suited to modelling chronic disease [30]. By utilising the ACE-Obesity policy model and the consistent approaches to determining cost-effectiveness adopted within the priority-setting study, the findings from this economic evaluation are comparable with the broader priority-setting study findings [22, 29]. The intervention-related changes to the distribution of risk factors (BMI, physical activity) on the incidence of disease related to that risk factor were estimated. Reduced incidence of diseases results in reductions in prevalence and disease-related mortality and morbidity. This leads to improved long term health outcomes and healthcare cost-savings [18–22]. Cohort-based modelling allowed health-related quality of life (HRQoL) and obesity-related health benefits, which are not present in early childhood, to be estimated assuming lingering BMI effects. For weight loss maintained into adulthood, population impact fractions (PIFs) were estimated [31] and used to estimate the consequences of a change in BMI on the incidence of nine obesity-related diseases (ischaemic heart disease, hypertensive heart disease, ischaemic stroke, diabetes, colorectal cancer, kidney cancer, breast cancer, endometrial cancer and osteoarthritis). PIFs were also used to estimate the consequences of a change in MET minutes on physical activity-related diseases (ischemic heart disease, stroke, type 2 diabetes, breast cancer and colon cancer) [32], and published adjustment factors were applied to avoid double-counting [33]. The base case analysis assumed maintenance of intervention effect over the lifetime.

The model used data from the Australian Health Survey 2011-12 and disease epidemiology from the Global Burden of Disease study (Table 1) [34, 35]. The change in risk was compared against the counterfactual, where the distributions in the 2010 Australian reference population remained unchanged. Results were presented as life years (LYs) gained, health adjusted life years (HALYs) gained, and healthcare cost-savings from diseases averted. HALYs were estimated by aggregating the population level changes to mortality and morbidity for each disease (using Global Burden of Disease disability weights [36] and the negative HRQoL impacts attributable to BMI in childhood) [37]. Incremental cost-effectiveness ratios (ICERs) were calculated and presented on a cost-effectiveness plane. Cost-effectiveness was determined using the threshold of AUD50,000 per HALY gained [38]. All modelling was undertaken in Microsoft Excel 2016. The model is available upon request to the corresponding author.

**Table 1:** Key modelling variables included in the economic evaluation

Parameters	Data source and assumptions
Intervention effect SB-I arm: BMI z-score (-0.14 (95%UI -0.26:-0.03) MET minutes per week (125 (95% UI 65–190) PA-I arm: BMI z-score (-0.13 BMI-z (95%UI -0.24:-0.03)	Normal distribution, using evidence of effectiveness from Salmon et al. [14] Base case analysis assumes maintenance of effect over the lifetime.
Total population estimates (population numbers, mortality rates, BMI distribution, PA levels)	Australian Bureau of Statistics [34, 39]
Disease epidemiology, disability weights	Salomon et al. 2012 [36]
Relative risks, total years of life lived with disability	Institute for Health Metrics and Evaluation [35] Murray et al. 2013 [40]
Relative risks of PA-related diseases by risk categories	Zapata-Diomedi et al. 2016 [41]
Disease healthcare costs	Australian Institute of Health and Welfare [42]
Health Price Index	Australian Institute of Health and Welfare [43]
Proportion of Grade 3 students in Government schools, Australia 2010	Australian Bureau of Statistics [17]
Number of children per class	Pert distribution* (min 10, most likely 23, max 30) using data from the Victorian Department of Education and Training [44]
Teacher salary cost	Gamma distribution using data on mean teacher salaries obtained from the Victorian Department of Education and Early Childhood [45]
Program Administrative and Support Services Officer cost, assumed for each Australian state and territory education department (*n* = 8)	Gamma distribution, ABS average weekly earnings Professional, Scientific and Technical Services full time adult average salary cost and 14% labour on-costs, from Government sources [46, 47]
Equipment costs	Project invoices, pert distribution (+/- 10%)

*Table notes:* * The Pert distribution is defined by the minimum, most likely and maximum values that a variable can take. The mean of the Pert distribution is the weighted average of the parameters (with four times the weight applied to the most likely value). 95% UI = 95% uncertainty interval. ABS = Australian Bureau of Statistics. AUD = Australian dollars. BMI-z = BMI z-score. BMI = body mass index. MET = metabolic equivalent task. PA = physical activity. UI = uncertainty interval

### Uncertainty and sensitivity analyses

Uncertainty analysis around key input parameters was conducted based on Monte Carlo simulation (2,000 iterations) using the Excel software add-in Ersatz (version 1.35) [48]. One-way sensitivity analysis tested the impact on results when all teacher preparation time data was included. Limited evidence exists on the long-term maintenance of the intervention effect, although the use of effect estimates at 30 months (i.e. after the 12 month tapered maintenance period) may suggest sustained changes to behaviour amongst intervention participants. One-way sensitivity analysis assumed the full decay of the intervention effect after 10 years [49], consistent with the findings from a review of similar studies [50].

Other factors considered important to decision-makers, including feasibility, sustainability, acceptability and equity impacts, are discussed alongside the cost-effectiveness results [23].

## Results

### Cost of the intervention

Completion rates of the time use diary varied by intervention group (Additional File 2). In total, 2,425 intervention activities were reported, covering five different categories of activities (Additional File 3).

The PA + SB-I intervention was the most costly intervention. Assuming class sizes based on data from the Victorian Department of Education and Training (Table 1) [44], the total cost per participant over 30 months was estimated to be AUD91 (95%UI AUD56-153), compared to AUD49 (95%UI AUD30-82) and AUD83 (95%UI AUD53-138) for the PA-I and SB-I interventions, respectively. This, however, suggests some economies of scale in delivery of the combined PA + SB-I intervention. The major driver of school costs was the extra time teachers needed to prepare delivery of *Transform-Us!* activities (Additional File 4). These preparation time costs comprised 70–88% of total costs borne by schools over the 30-month period. Teachers in the PA-I group reported less teacher time planning activities in both the intervention and maintenance phase, leading to lower overall costs as compared to the SB-I and SB + PA-I arms.

### Cost-effectiveness

#### Modelled cost-effectiveness in the trial population

Modelled cost-effectiveness analysis of the trial population suggests relatively small health benefits (HALYs saved) and healthcare cost-savings for participants in PA-I and SB-I if maintenance of the effect is assumed over the lifetime (Table 2). The PA-I and SB-I groups were both dominant (i.e., cost-saving and health-promoting). The cost-effectiveness of the PA + SB-I group was not estimated, given that the intervention effects on BMI and sedentary time were non-significant at 30 months [14].

**Table 2:** Modelled trial population cost-effectiveness results, over the lifetime

Metric	PA-I	SB-I
**Mean modelled decrease in BMI**	Males: 0.55 kg/m^2^ (95% UI 0.12–0.99) Females: 0.43 kg/m^2^ (95% UI 0.09–0.76)	Males: 0.60 kg/m^2^ (95% UI 0.12–1.06) Females: 0.47 kg/m^2^ (95% UI 0.09–0.82)
**Mean MET minutes gained per week**	N/A	125 (95% UI 65–190)
**Total LYs gained over lifetime**	78 (95% UI 15–143)	76 (95% UI 12–148)
**Total HALYS saved over lifetime**	134 (95% UI 26–251)	140 (95% UI 22–259)
**Total healthcare cost-savings over lifetime**	AUD1.4 M (95% UI AUD276 017-2.6 M)	AUD1.5 M (95% UI AUD246 485-2.8 M)
**Total intervention costs**	AUD19 759 (95% UI AUD19 452 − 20 094)	AUD32 380 (95% UI AUD32 174 − 32 579)
**Total net cost** ^**a**^	AUD-1.4 M (95% UI AUD-2.6 M to -256,069)	AUD-1.5 M (95% UI AUD-2.8 M to -214,066)
**Net cost per HALY saved (ICER)** ^**a**^	AUD-10 442 (95% UI AUD-8 883 to -11,863)	AUD-10 672 (95% UI AUD-8 772 to -12 329)
**Overall result**	Dominant^b^ (95% UI dominant-dominant)	
**Probability of cost-effectiveness**	99.5%	99.2%

*Table notes:*^a^ Negative total net costs equate to cost-savings. ^b^ Dominant interventions result in health gains and cost-savings. ^c^ The willingness-to-pay threshold for this analysis is AUD50,000 per HALY. Costs are presented in 2010 Australian dollars. 95% UI = 95% uncertainty interval based on 2,000 simulations. AUD = Australian dollars. BMI = body mass index. HALYs = Health adjusted life years. ICER = Incremental cost-effectiveness ratio. Kg = kilograms. LYs = life years. m = metres. M = million. MET = metabolic equivalent task. N/A = not applicable. PA-I = physical activity intervention. SB-I = sedentary behaviour intervention

#### Cost-effectiveness results from extrapolation

Extrapolating the PA-I and SB-I intervention groups to the Australian population of Year 3 children in Australian Government schools (*n* = 184,547 children) led to more significant health benefits and healthcare cost-savings, assuming maintenance of effect over the lifetime (Table 3).

**Table 3:** Cost-effectiveness results, extrapolating PA-I and SB-I to the Australian population of Year 3 children in Government schools, over the lifetime

Metric	PA-I	SB-I
**Total LYs gained over lifetime**	36 588 (95% UI 9 070 to 65 519)	35 643 (95% UI 7 009 to 65 711)
**Total HALYS saved over lifetime**	60 780 (95% UI 15 007 to 109 413)	61 125 (95% UI 11 770 to 111 250)
**Total healthcare cost-savings over lifetime**	AUD641M (95% UI AUD165M to 1.1B)	AUD654M (95% UI AUD126M to 1.2B)
**Total intervention costs**	AUD10M (95% UI AUD7M to 15 M)	AUD15M (95% UI AUD10M to 26 M)
**Total net cost** ^**a**^	AUD-631 M (95% UI AUD-1.1B to -155 M)	AUD-638 M (95% UI AUD-1.2B to -110 M)
**Net cost per HALY saved (ICER)** ^**a**^	AUD-10 374 (95% UI AUD-8 934 to -11,674)	AUD-10 444 (95% UI AUD-8 721 to -11 854)
**Overall result**	Dominant^b^ (95% UI Dominant to Dominant)	
**Probability of cost-effectiveness** ^**c**^	99.4%	99.2%

*Table notes:*^a^ Negative total net costs equate to cost-savings. ^b^ Dominant interventions result in health gains and cost-savings. ^c^ The willingness-to-pay threshold for this analysis is AUD50,000 per HALY. Costs are presented in 2010 Australian dollars. 95% UI = 95% uncertainty interval based on 2,000 simulations. AUD = Australian dollars. BMI = body mass index. HALYs = Health adjusted life years. ICER = Incremental cost-effectiveness ratio. LYs = life years. M = million. MET = metabolic equivalent task. N/A = not applicable. PA-I = physical activity intervention. SB-I = sedentary behaviour intervention

**Fig. 1:**
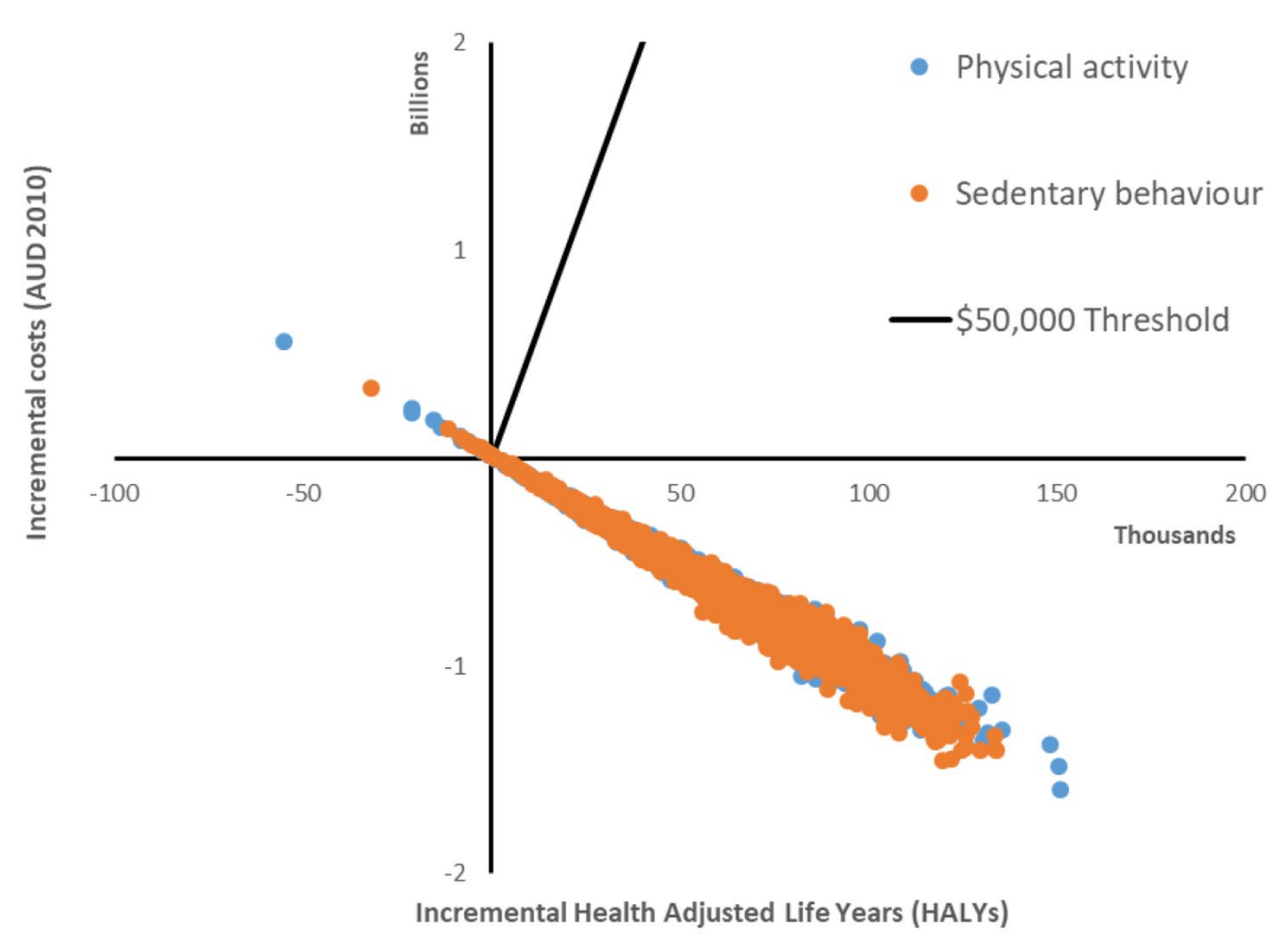
Cost-effectiveness plane, extrapolating costs and effects to the Australian population of Grade 3 children in Government schools. *Figure notes:* AUD = Australian dollars. Parameter uncertainty is demonstrated through probabilistic sensitivity analysis

Figure 1 presents the cost-effectiveness plane when extrapolating the costs and effects of the intervention groups to the population of Year 3 children in Australian Government schools. Both the PA-I and SB-I interventions represent value for money as obesity prevention measures if effects are sustained (probability of cost-effectiveness 99%).

#### Sensitivity analysis results

**Fig. 2:**
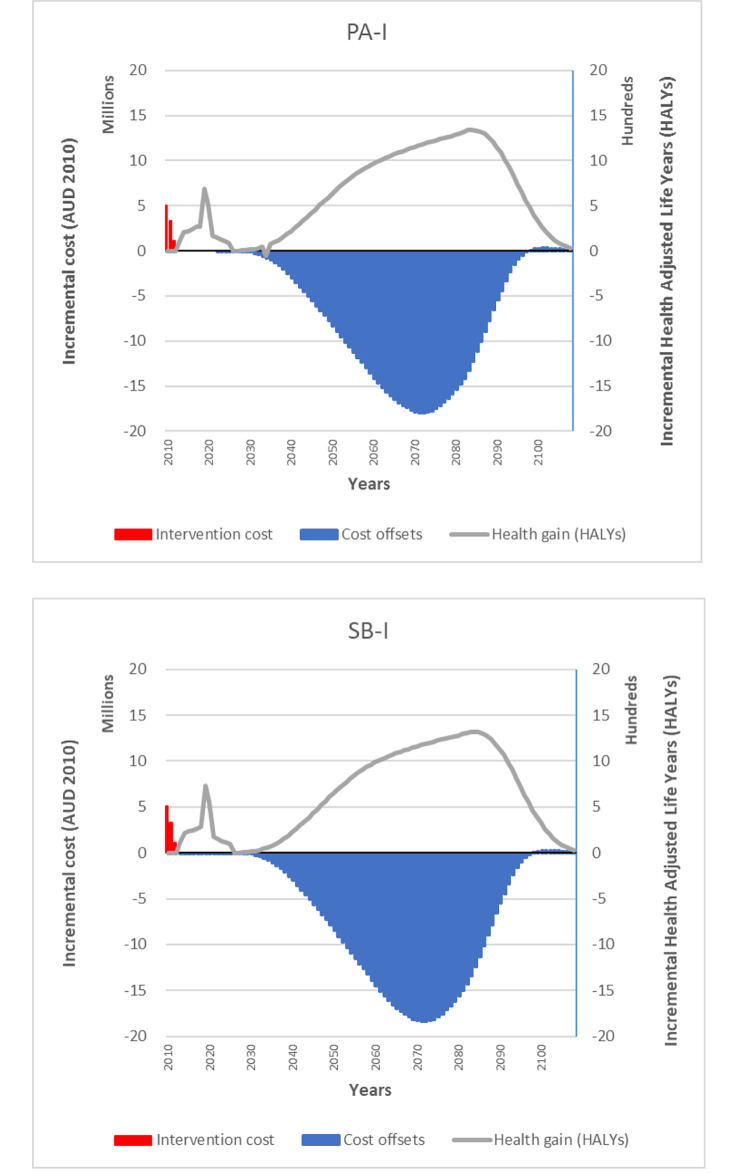
Intervention costs, cost offsets and health gains over time, PA-I and SB-I interventions. *Figure notes:* AUD = Australian dollars. HALYs = health-adjusted life years. PA-I = physical activity intervention group. SB-I = sedentary behavior intervention group

Although incorporating outlier teacher preparation time data resulted in more costly interventions (PA-I: AUD74 (95%UI AUD48-120) over 30 months; SB-I: AUD148 (95%UI AUD93-243) over 30 months), both interventions remained dominant (PA-I: ICER − 10,299 (95%UI -8,652:-11,617); SB-I: -10,244 (95%UI -7,844:-11,730). Figure 2 demonstrates the intervention costs, cost offsets and health gains over the lifetime of the cohort for the SB-I and PA-I intervention groups. Whilst the costs of the intervention are incurred upfront, the benefits of the intervention in terms of healthcare cost-offsets of diseases averted do not start to accrue until at least 20 years in the future and peak at around the time that the cohort of Year 3 children are 70 years of age. Health benefits related to an improvement in health-related quality of life in childhood occur in the short-term, long before the health benefits related to the prevention of chronic diseases occur (Fig. 2). If we assume that the intervention effect decays to zero after 10 years, the health benefits of the PA-I and SB-I intervention groups are more modest (PA-I: 2,479 HALYs saved (95%UI 558-4,333); SB-I: 2,650 HALYs saved (95%UI 628-4,579)). However, the intervention is still considered cost-effective (mean ICER PA-I: $4,056 per HALY gained, SB-I: $5,788 per HALY gained).

An analysis of other issues relevant to intervention implementation demonstrates that the *Transform-Us!* intervention would be feasible and acceptable to multiple stakeholders, although would require an on-going commitment to funding if the program was to be sustained (Table 4).

**Table 4:** Intervention implementation considerations

Implementation consideration		Overall rating
Equity	The intervention is delivered in primary schools, and therefore is more likely to be equitable assuming funding is available to resource the program.	*Positive*
	Medium certainty of BMI effect, objectively measured in one high quality RCT.	Medium
Strength of evidence	Medium certainty of SB effect, device measured in one high quality RCT.	Medium
Acceptability	Government: Federal and State Governments are generally supportive of programs designed to improve the health of school students. The intervention may help to fulfill the criteria for several Australian Curriculum guidelines focused on health and physical education.	*High*
	Industry: The intervention could provide valuable resources for teachers and schools to meet the relevant guidelines. Anecdotally, teachers and schools were generally receptive to the intervention, but listed time constraints and competing demands on their time as potential barriers to program delivery.	*High*
	Public: The general public is likely to be supportive of programs that improve the health of school children. Anecdotally, the intervention was positively received by parents and children.	*High*
Feasibility	Interventions delivered in the school environment are feasible.	*High*
Sustainability	Interventions delivered in the school environment are sustainable with ongoing support and appropriate funding.	Medium
Other considerations	The intervention may have a positive effect on the families of children who participate, however no evidence of this effect is currently available.	

Note: BMI: body mass index. RCT: randomized controlled trial. SB: sedentary behavior.

Table notes: Adapted from Ananthapavan et al [51]

## Discussion

The *Transform-Us!* intervention aimed to improve physical activity and reduce sedentary behaviour in primary school children in Melbourne, Australia. Both the PA-I and SB-I intervention groups demonstrated statistically significant effects for reducing BMI z-score at 30 months, with the SB-I intervention arm also achieving a statistically significant effect for reducing device-measured sedentary time [14]. Because the *Transform-Us!* intervention was not resource intensive and was relatively low cost, intervention groups that were able to demonstrate even relatively small BMI-related outcomes were considered cost-effective as obesity prevention measures assuming maintenance of effect for at least ten years.

*Transform-Us!* was low cost because the intervention design maximized reach and presented economies of scale by training each classroom teacher to deliver the intervention to a full class of Year 3 children. The key cost driver for schools was the teacher preparation time to deliver lessons to students. The extra preparation was typically less than 10 min per activity and although this additional preparation time did not decline over the 30-month trial period, it might be expected that if these activities were fully embedded into the school curriculum and became routine that preparation time would decrease, further reducing intervention costs.

The review by Oosterhoff et al. noted that methodological differences in economic evaluations of school-based lifestyle interventions limited comparability of findings [10]. While there are methodological differences between previous studies and this trial that make a direct comparison problematic, the results demonstrate the potential value for money of the *Transform-Us!* physical activity and sedentary behaviour interventions, when compared to other obesity prevention interventions that have been undertaken in Australian school or community settings (Additional File 5). When compared to interventions estimated in an Australian priority-setting study that used consistent methodologies and where comparability between the results from this study are valid, the *Transform-Us!* PA and SB interventions ranked as the seventh and eighth most cost-effective interventions (out of 16 interventions) [22]. 

Study strengths included the long length of the trial and the use of effectiveness estimates achieved after a sustained maintenance period (i.e. at 30 months). It should, however, be noted that the determinants of physical activity maintenance are complex, and there is mixed evidence on the tracking of physical activity from childhood to adolescence and adulthood (for example, [50, 52–54]). This is a significant area for future research. We also undertook detailed micro-costing of the intervention and extrapolated intervention costs and effects using a modelling framework, taking into account potential consequences on costs and benefits in the long-run and if the intervention were scaled up to a national context [55]. Limitations included the 36% response rate for participation in the evaluation of the intervention and measurement of outcomes [14], and the varied teacher diary response rates between intervention groups (Additional File 2). We were limited in undertaking a societal perspective as per health economics guidance [15], as data to inform the broader costs and benefits of the intervention (for example, productivity effects) were not available. In addition, the costs of scaling up the intervention to delivery across all government schools were estimated and therefore may not be reflective of actual implementation costs. This is an area for more exploration; in particular, the impacts of implementation at scale on overall cost-effectiveness warrant further research. A significant area for future work is a comprehensive assessment of how to better incorporate considerations of scale into health economic analysis, particularly important for building the evidence of cost-effectiveness for preventive public health interventions at the population level. While our analyses did not include costs associated with intervention reach and adoption in the school setting, it could be hypothesized that incorporation of the intervention into the Australian curriculum could help to minimize such costs. Our base case results assumed lifetime maintenance of intervention effect and the only shorter-term benefit of obesity prevention in children captured within our analyses is the potential impact on health-related quality of life [56]. We have not included any health benefits or costs related to potential improvements in sleep apnea, asthma, blood pressure or other health issues that may be experienced within the childhood years [57] as a result of the intervention. Our results may therefore under-estimate intervention cost-effectiveness. Further incorporation of health costs and consequences within the child and adolescent timeframes is an area for future work [10]. 

## Conclusion

The *Transform-Us!* physical activity and sedentary behaviour interventions have significant potential for cost-effectiveness as obesity prevention measures and represent good investments in the health of Australian children. Our analysis demonstrates that both the PA-I and SB-I intervention groups were cost-saving, with the potential to reduce the burden of chronic disease associated with obesity and physical inactivity if the effect can be sustained over time. More research is required into the health benefits and healthcare cost-savings that accrue within the child and adolescent years, to further strengthen the estimates of cost-effectiveness. This study highlights the significant potential for improved health and reduced healthcare spending if children can attain and maintain a healthy weight into adulthood.

### Electronic supplementary material

Below is the link to the electronic supplementary material.

Supplementary Material 1: Consolidated Health Economic Evaluation Reporting Standards (CHEERS) checklist items reported in the economic evaluation of the *Transform-Us*! RCT.

Supplementary Material 2: Average return rate of the teacher diary during the intervention period, PA-I and SB-I interventions.

Supplementary Material 3: Total number of *Transform-Us!* activities costed using returned teacher diaries over the 30 month trial period.

Supplementary Material 4: Contribution of major cost categories to 30-month intervention costs.

Supplementary Material 5: Comparison of results from the Transform-Us! intervention with school-based ACE-Obesity [23] studies targeting PA or sedentary behaviours.

Supplementary Material 6

## Abbreviations

ACE: Assessing Cost-Effectiveness
AUD: Australian dollars
B: Billions
BMI: Body mass index
BMI: z-Body mass index z-score
C: Usual curriculum control
cRCT: Cluster randomized controlled trial
HALYs: Health-adjusted life years
HRQoL: Health-related quality of life
ICER: Incremental cost-effectiveness ratio
Kg: Kilograms
LY: Life years
m: Metres
M: Millions
MET: Metabolic equivalent task
PA: I-Intervention targeting increases in physical activity
PA + SB: I-Combined physical activity and sedentary behavior intervention
PIF: Population impact fraction
RCT: Randomized controlled trial
SB: I-Intervention targeting decreases in sedentary behavior
UI: Uncertainty interval
