# Supplementary material for: Cost-effectiveness of reducing children’s sedentary time and increasing physical activity at school: the Transform-Us! intervention
Source: Int J Behav Nutr Phys Act. 2024 Feb 12;21:15. doi: 10.1186/s12966-024-01560-3 (PMC10860323; doi:10.1186/s12966-024-01560-3)
Supplement: Supplementary file 1 — Supplementary Material 1: Consolidated Health Economic Evaluation Reporting Standards (CHEERS) checklist items reported in the economic evaluation of the Transform-Us! RCT. [file 12966_2024_1560_MOESM1_ESM.docx]

**Additional File 1 –**

**Consolidated Health Economic Evaluation Reporting Standards (CHEERS) checklist (1) items reported in the economic evaluation of the *Transform-Us*! RCT**

| **CHEERS checklist item** | **Economic evaluation of the Transform-Us! study** | **Page number** |
| --- | --- | --- |
| Economic evaluation undertaken | Modelled CUA | Title  Abstract  8 |
| Context of the study | Limited evidence exists on the cost-effectiveness of interventions aimed at improving physical activity and reducing sedentary behaviour in children. | 4-5 |
| Target population and sub-groups | 1,606 children aged 8-9 years (Grade 3) that attended primary schools within a 50km radium of Melbourne, Australia. Intervention cost and effect was then extrapolated to the Australian population of Grade 3 children in Government schools (69% of Australian males aged 8 years, 68% of Australian females aged 8 years)(2). | 5  8 |
| Setting and location | Primary school setting, Australia | 5 |
| Study perspective | Public payer | 6 |
| Comparators | Usual practice (no intervention) | 6 |
| Time horizon | Lifetime or 100 years | 6 |
| Discount rate | 3% | 6 |
| Choice of health outcomes | Based on detailed study results published in this issue (Salmon et al.). Statistically significant intervention effects (comparison between each experimental group and the control group) related to sedentary time and BMI at 30 months.  BMIz at 30 months; sedentary time (minutes per weekday) at 30 months | 8  9 |
| Measurement of effectiveness | Based on detailed study results (Salmon et al.). Statistically significant intervention effects (comparison between each experimental group and the control group) related to sedentary time and BMI at 30 months. | 8 |
| Measurement and valuation of preference-based outcomes | Based on the literature: Chen G, Ratcliffe J, Olds T, Magarey A, Jones M, Leslie E. BMI, health behaviors, and quality of life in children and adolescents: A school-based study. Pediatrics. 2014;133(4). | 9  10 |
| Estimating resources and costs | Pathway analysis, comprehensive within-trial costing. Major cost categories were defined as related to teacher time, purchase of school equipment and development and dissemination of newsletters. Unit costing undertaken using trial data for the 30 month intervention and published sources. Given intervention scale-up, program administration costs (1 FTE Administrative and Support Services Officer per Australian state and territory (n=8)) were assumed using published sources. | 7  8 |
| Currency, price date and conversion | AUD 2010 | 8 |
| Choice of model | Proportional multi-state lifetable model, used for priority-setting study conducted in Australia (the ACE-Obesity Policy model)(3). | 8  9 |
| Assumptions | Maintenance of intervention effect; useful life of equipment in schools; MET value. | 7,8, 9,13 |
| Analytic methods | Generalised linear mixed models were used to determine intervention efficacy compared to control.  A proportional multi-state lifetable model was used to conduct the CUA (3). | 8  9 |
| Uncertainty and sensitivity | Uncertainty analysis around key input parameters was conducted based on a Monte Carlo simulation (2,000 iterations) using the Excel software add-in Ersatz (version 1.35) (4). Sensitivity analyses were conducted: assuming the full decay of the intervention effect after 10 years; and including all teacher time cost data. | 10 |
| Source of funding | National Health & Medical Research Council (NHMRC) of Australia Project Grant (533815); Diabetes Australia Research Trust. The funders played no role in the design of the study, the collection, analysis or interpretation of the data, in the writing of the paper, or the decision to submit for publication. At the time of this study VB and MM were researchers with the National Health and Medical Research Council (NHMRC) funded Centre for Research Excellence (CRE) in Obesity Policy and Food Systems (1041020) and the CRE in the Early Prevention of Obesity in Childhood (CRE EPOCH; 1101675). VB was supported by an Alfred Deakin Postdoctoral Research Fellowship. JS is supported by a NHMRC Leadership Level 2 Fellowship (1176885). DWD is supported by a NHMRC Senior Research Fellowship (1078360) and in part by the Victorian Government’s OIS Program. KDH is supported by an Australian Research Council Future Fellowship (FT130100637). NR is supported by a Future Leader Fellowship from the National Heart Foundation of Australia (101895). At the time of the study, LA was supported by an Alfred Deakin Postdoctoral Research Fellowship. EC was supported by an Australian Research Council Future Fellowship (FT140100085). | 20 |
| Conflict of interest | No conflict of interest declared |  |

*Table notes*: AUD= Australian dollars. BMI= body mass index. CRE= Centre for Research Excellence. CUA= cost-utility analysis. FTE: full-time equivalent. n/a=not applicable. MET= metabolic equivalent task. NHMRC= National Health and Medical Research Council. PA= physical activity.

**REFERENCES**

1. Husereau D, Drummond M, Augustovski F, de Bekker-Grob E, Briggs AH, Carswell C, et al. Consolidated Health Economic Evaluation Reporting Standards 2022 (CHEERS 2022) statement: updated reporting guidance for health economic evaluations. BMJ. 2022;376:e067975.

2. Australian Bureau of Statistics. 4221.0 - Schools, Australia, 2010. Canberra, Australia: ABS; 2011.

3. Ananthapavan J, Sacks G, Brown V, Moodie M, Nguyen P, Barendregt J, et al. Priority-setting for Obesity Prevention - the Assessing Cost-Effectiveness of Obesity Prevention Policies in Australia (ACE-Obesity Policy) study. PLOS One. 2020;15: e0234804.

4. EpiGear International. Ersatz Brisbane, Australia: EpiGear International; 2016 [Available from: <http://www.epigear.com/index_files/ersatz.html>.

5. Carter R, Moodie M, Markwick A, Magnus A, Vos T, Swinburn B, et al. Assessing cost-effectiveness in obesity (ACE-obesity): an overview of the ACE approach, economic methods and cost results. BMC Public Health. 2009;9:419.

6. Australian Bureau of Statistics. Consumer Price Index, Australia. Cat No. 6401.0 2010 [Available from: <http://www.abs.gov.au/AUSSTATS/abs@.nsf/allprimarymainfeatures/C8BF4F29155866E0CA2578790014B817?opendocument>.

7. Victorian Government Department of Human Services. ACE-Obesity: Assessing Cost-effectiveness of Obesity Interventions in Children and Adolescents. Summary of Results. Melbourne, Australia; 2006.

8. Moodie M, Haby MM, Swinburn B, Carter R. Assessing cost-effectiveness in obesity: active transport program for primary school children--TravelSMART Schools Curriculum program. Journal of Physical Activity & Health. 2011;8(4):503-15.

9. Moodie M, Haby M, Galvin L, Swinburn B, Carter R. Cost-effectiveness of active transport for primary school children - Walking School Bus program. The International Journal of Behavioral Nutrition and Physical Activity. 2009;6:63.
