# Supplementary material for: Cost-effectiveness of reducing children’s sedentary time and increasing physical activity at school: the Transform-Us! intervention
Source: Int J Behav Nutr Phys Act. 2024 Feb 12;21:15. doi: 10.1186/s12966-024-01560-3 (PMC10860323; doi:10.1186/s12966-024-01560-3)
Supplement: Supplementary file 2 — Supplementary Material 2: Average return rate of the teacher diary during the intervention period, PA-I and SB-I interventions. [file 12966_2024_1560_MOESM2_ESM.docx]

**Additional File 2 –**

**Average return rate of the teacher diary during the intervention period, PA-I and SB-I interventions**

| **Intervention group** | **18-month intervention** | | **12-month follow up** |
| --- | --- | --- | --- |
|  | **2010 (%)** | **2011 (%)** | **2012 (%)** |
| **PA-I** | 55 | 37 | 74 |
| **SB-I** | 47 | 55 | 26 |

*Table notes:* PA-I= physical activity group. SB-I= sedentary behavior group.
