# Supplementary material for: Cost-effectiveness of reducing children’s sedentary time and increasing physical activity at school: the Transform-Us! intervention
Source: Int J Behav Nutr Phys Act. 2024 Feb 12;21:15. doi: 10.1186/s12966-024-01560-3 (PMC10860323; doi:10.1186/s12966-024-01560-3)
Supplement: Supplementary file 3 — Supplementary Material 3: Total number of Transform-Us! activities costed using returned teacher diaries over the 30 month trial period. [file 12966_2024_1560_MOESM3_ESM.docx]

**Additional File 3 –**

**Total number of *Transform-Us!* activities costed using returned teacher diaries over the 30 month trial period**

| **Intervention group** | **Key messages** | **Standing lessons** | **Active breaks** | **Homework tasks** | **Recess/Lunch games** | **Total** |
| --- | --- | --- | --- | --- | --- | --- |
| **PA-I** | 154 | n/a | n/a | 65 | 270 | 489 |
| **SB-I** | 52 | 216 | 218 | 19 | n/a | 505 |
| **PA+SB-I** | 169 | 465 | 553 | 51 | 193 | 1,431 |
| **Total** | **375** | **681** | **771** | **135** | **463** | **2,425** |

*Table notes:* PA-I= physical activity intervention group. SB-I= sedentary behavior intervention group. PA+SB-I= combined intervention group. n/a= the intervention group was not required to undertake the activity
