# Supplementary material for: Cost-effectiveness of reducing children’s sedentary time and increasing physical activity at school: the Transform-Us! intervention
Source: Int J Behav Nutr Phys Act. 2024 Feb 12;21:15. doi: 10.1186/s12966-024-01560-3 (PMC10860323; doi:10.1186/s12966-024-01560-3)
Supplement: Supplementary file 4 — Supplementary Material 4: Contribution of major cost categories to 30-month intervention costs. [file 12966_2024_1560_MOESM4_ESM.docx]

**Additional File 4 – Contribution of major cost categories to 30-month intervention costs**

| **Cost contribution category** | **Intervention group** | | |
| --- | --- | --- | --- |
|  | **PA-I** | **SB-I** | **SB+PA-I** |
| Teacher time costs, intensive phase | 67% | 70% | 67% |
| Teacher time costs, maintenance phase | 3% | 18% | 13% |
| Equipment costs | 18% | 5% | 13% |
| Ongoing implementation | 12% | 7% | 6% |
| Total | 100% | 100% | 100% |

*Table notes:* PA-I= physical activity intervention. SB-I= sedentary behaviour intervention. SB+PA-I= combination physical activity and sedentary behaviour intervention.
