# Supplementary material for: Cost-effectiveness of reducing children’s sedentary time and increasing physical activity at school: the Transform-Us! intervention
Source: Int J Behav Nutr Phys Act. 2024 Feb 12;21:15. doi: 10.1186/s12966-024-01560-3 (PMC10860323; doi:10.1186/s12966-024-01560-3)
Supplement: Supplementary file 5 — Supplementary Material 5: Comparison of results from the Transform-Us! intervention with school-based ACE-Obesity [23] studies targeting PA or sedentary behaviours. [file 12966_2024_1560_MOESM5_ESM.docx]

**Additional File 5- Comparison of results from the Transform-Us! intervention with school-based ACE-Obesity (5) studies targeting PA or sedentary behaviours**

Several other Australian school-based interventions have been evaluated for their economic credentials as obesity prevention interventions as part of the Assessing Cost-Effectiveness (ACE) Obesity body of work (5), and reported in AUD2001 prices. To facilitate comparison, relevant costs from these studies were adjusted to the 2010 reference year using the Australian Consumer Price Index (6).

| **Intervention** | **Results from economic evaluation** | **Intervention cost per child**  **(AUD2010 prices)** |
| --- | --- | --- |
| *TransformUs!* | | |
| PA-I | Dominant^a^ | AUD49 |
| SB-I | Dominant^a^ | AUD81 |
| *ACE-Obesity studies* | | |
| Multi-faceted program, including education to improve nutrition and increase PA, with an active PE component (7) | Dominant^a^ | AUD608^b^ |
| Multi-faceted program targeted at overweight and obese children (7) | Dominant^a^ | AUD166^b^ |
| Education program to reduce TV viewing (7) | Dominant^a^ | AUD133^b^ |
| TravelSmart Schools program (8) | Not cost-effective | AUD64^b^ |
| Walking School Bus program (9) | Not cost-effective | AUD3,737^b^ |

*Table notes:* ^a^ Dominant interventions result in health gains and cost-savings. ^b^ Study base year prices adjusted to Australian dollar (AUD) 2010 prices using the Australian Consumer Price Index (6). PA= physical activity. PE= physical education. PA-I= physical activity intervention. SB-I= sedentary behavior intervention.

**REFERENCES**

1. Husereau D, Drummond M, Augustovski F, de Bekker-Grob E, Briggs AH, Carswell C, et al. Consolidated Health Economic Evaluation Reporting Standards 2022 (CHEERS 2022) statement: updated reporting guidance for health economic evaluations. BMJ. 2022;376:e067975.

2. Australian Bureau of Statistics. 4221.0 - Schools, Australia, 2010. Canberra, Australia: ABS; 2011.

3. Ananthapavan J, Sacks G, Brown V, Moodie M, Nguyen P, Barendregt J, et al. Priority-setting for Obesity Prevention - the Assessing Cost-Effectiveness of Obesity Prevention Policies in Australia (ACE-Obesity Policy) study. PLOS One. 2020;15: e0234804.

4. EpiGear International. Ersatz Brisbane, Australia: EpiGear International; 2016 [Available from: <http://www.epigear.com/index_files/ersatz.html>.

5. Carter R, Moodie M, Markwick A, Magnus A, Vos T, Swinburn B, et al. Assessing cost-effectiveness in obesity (ACE-obesity): an overview of the ACE approach, economic methods and cost results. BMC Public Health. 2009;9:419.

6. Australian Bureau of Statistics. Consumer Price Index, Australia. Cat No. 6401.0 2010 [Available from: <http://www.abs.gov.au/AUSSTATS/abs@.nsf/allprimarymainfeatures/C8BF4F29155866E0CA2578790014B817?opendocument>.

7. Victorian Government Department of Human Services. ACE-Obesity: Assessing Cost-effectiveness of Obesity Interventions in Children and Adolescents. Summary of Results. Melbourne, Australia; 2006.

8. Moodie M, Haby MM, Swinburn B, Carter R. Assessing cost-effectiveness in obesity: active transport program for primary school children--TravelSMART Schools Curriculum program. Journal of Physical Activity & Health. 2011;8(4):503-15.

9. Moodie M, Haby M, Galvin L, Swinburn B, Carter R. Cost-effectiveness of active transport for primary school children - Walking School Bus program. The International Journal of Behavioral Nutrition and Physical Activity. 2009;6:63.
