# Supplementary material for: Cost-effectiveness of reducing children’s sedentary time and increasing physical activity at school: the Transform-Us! intervention
Source: Int J Behav Nutr Phys Act. 2024 Feb 12;21:15. doi: 10.1186/s12966-024-01560-3 (PMC10860323; doi:10.1186/s12966-024-01560-3)
Supplement: Supplementary file 6 — Supplementary Material 6 [file 12966_2024_1560_MOESM6_ESM.docx]

**Manuscript ID**

'IJBN-D-23-00615 - Cost-effectiveness of an intervention to reduce children’s sedentary time and increase physical activity: the Transform-Us! cluster RCT'

| **Response to reviewer’s comments** | | | |
| --- | --- | --- | --- |
| The authors thank the reviewers for their time taken to provide valuable feedback, and for the opportunity to resubmit the paper addressing their comments. | | | |
| Reviewer #1 | | Author’s response | Changes to document |
| This article is a well-written health economic study documenting the estimated costs and cost-effectiveness of school-based strategies to tackle childhood obesity. It is interesting and would make a good addition to the literature. The methods used are clear and transparently reported, making good use of supplementary files. A significant amount of work has gone into this piece and I appreciate the vast amount of information needed to be reduced into the word limits for a manuscript. Overall, I believe this is an important contribution to the literature.  Some mostly minor comments are as follows: | | | |
| 1 | Although the authors emphasize in the title that the study is based on an RCT, it is not a within-trial economic evaluation so it may be misleading to use 'RCT' in the title (also because it is an acronym) rather than a modelling-based analysis which it more accurately is. | We agree and have amended the title for more clarity. | Title change:  Cost-effectiveness of ~~an intervention to~~ reducing children’s sedentary time and increasing physical activity at school: the Transform-Us! ~~cluster RCT~~ intervention |
| 2 | Abstract Results: could the time horizon be added here as it is difficult to interpret the metrics without 'over the cohorts lifetime' or similar. | We have added this detail into the abstract. | Abstract, Methods:  A validated multiple-cohort lifetable model (ACE-Obesity Policy model) estimated the obesity and physical activity-related health outcomes and healthcare cost-savings over the cohort’s lifetime from the public-payer perspective, assuming the intervention was delivered to all 8-9 year old children attending Australian Government primary schools. |
| 3 | The last sentence of the Results in the Abstract: I wonder whether that should be removed because it raises the question of why the combined arm did not show effectiveness when effectiveness/CEA was shown in each of the two separate arms. | We have removed this sentence from the abstract. | Abstract, Results:  ~~Cost-effectiveness of the combined PA+SB-I intervention was not estimated because there were no statistically significant intervention effects for either BMI or sedentary time.~~ |
| 4 | Background - page 4, line 86, should education resources be mentioned here too as this is key for implementation. | We have added this detail into the manuscript. | Introduction, line 95:  Given the multitude of competing demands for both health and education resources, it is not only important that these interventions are effective, but they must also represent “good value for money”. |
| 5 | Page 4, Line 89, a systematic review with 23 studies is a lot of economic studies. What were their findings? | We have added more text to clarify this sentence. We have also added another sentence, summarising the findings from the systematic review. We have also further strengthened this point in the discussion section, by adding text re the comparability of findings from this study with others from the literature. | Introduction, lines 97-103:  To date, limited economic evidence for school-based interventions targeting physical activity or sedentary behaviours has been published, with a recent systematic review of school-based lifestyle interventions identifying ~~only~~ 23 studies in total (18 of which included physical activity-related intervention components).(10) The authors noted that heterogeneity in the measurement, valuation and extrapolation of costs and outcomes limited comparability of findings.(10) This limited comparability may impact the usefulness of economic evaluations of school-based interventions in informing the allocation of public budgets.(10)  Discussion, lines 405-406:  The review by Oosterhoff et al. noted that methodological differences in economic evaluations of school-based lifestyle interventions limited comparability of findings.(10) While there are methodological differences between previous school-based studies and this trial that make a direct comparison problematic, the results demonstrate the potential value for money of the *Transform-Us!* physical activity and sedentary behaviour interventions, when compared to other obesity prevention interventions that have been undertaken in Australian school or community settings (Additional File 5). When compared to interventions estimated in an Australian priority-setting study that used consistent methodologies and where comparability between the results from this study are valid, the *Transform-Us!* PA and SB interventions ranked as the seventh and eighth most cost-effective interventions (out of 16 interventions).(22) |
| 6 | Page 4, line 94, when were the 30 month follow-ups completed - it seems like this was 2012 which is a very long time ago. Why is there such a long gap for the main results to be published? | The investigators experienced significant delays in analysing the plasma outcomes from the RCT and wished to make sure that the main findings were published before the economic evaluation (published in March 2023, see reference (14)). | No changes made. |
| 7 | Methods - page 8, measurement of effectiveness, I think too much text is spent (lines 180-189) on the measures here and what I would have rather seen were the actual findings/reductions in BMI or MET changes at 30 months. These are inputs to the model coming directly from the trial and trial paper so additional detail can be in a supplementary file or refer readers to trial paper. | Thank you for this comment. We had previously provided the intervention effects in the Results section, under the heading ‘Effectiveness’. We have now moved this detail to the Methods section, as per your suggestion. We have opted to keep the detail on the measures in the methods section, as we feel that it is necessary to inform the reader on a key input parameter to our analysis. | Methods, line 207-216:  Measures of intervention effectiveness were based on detailed published study results.(14) Intervention effects (comparison between each experimental group and the control group) related to sedentary time and BMI at 30 months were assessed for statistical significance (p-value <0.05). Sedentary time was measured using accelerometry (ActiGraph GT3X (Pensacola, FL). Children’s height and weight were measured by trained research staff and used to estimate BMI z-score. ~~Full reporting on measurement of intervention effectiveness has been published previously.(11,13,14)~~  There was a statistically significant intervention effect among participants in the SB-I arm for BMI z-score at 30 months (-0.14 BMI-z (95%UI -0.26:-0.03)), as well as for sedentary time (-62.8 minutes per weekday (95%UI -92.0:-33.9)) compared to the control group. There was a statistically significant intervention effect among participants in the PA-I arm for BMI z-score (-0.13 BMI-z (95%UI -0.24:-0.03), but no statistically significant intervention effect on sedentary time. Participants in PA+SB-I did not report a statistically significant intervention effect for sedentary time or BMI z-score. Full reporting on measurement of intervention effectiveness has been published.(11,13,14)  Results, line 300:  Moved “Effectiveness” section from Results to Methods (red text above) |
| 8 | Page 9, line 191, On the other hand, the multi-state lifetime model has one sentence on it with little description of rationale for the type of model, validation undertaken or how it was constructed and what stakeholders were involved. Currently, the description doesn't meet the CHEERs checklist for modelling work. | Thank you for this comment. We have added additional detail on the description and rationale for the model. We have also added a sentence re the availability of the model. References to publications providing more detail are given in the text. | Methods, line 226-259:  A proportional multi-state lifetable Markov cohort model (the ACE-Obesity Policy model) was used to estimate cost-effectiveness.(18-22,29) The validated ACE-Obesity Policy model was developed as part of a large priority-setting study undertaken in Australia from 2012-2018.(18-22,29) Markov modelling is widely used in economic evaluation, and is particularly suited to modelling chronic disease.(30) By utilising the ACE-Obesity policy model and the consistent approaches to determining cost-effectiveness adopted within the priority-setting study, the findings from this economic evaluation are comparable with the broader priority-setting study findings.(22,29) The intervention-related changes to the distribution of risk factors (BMI, physical activity) on the incidence of disease related to that risk factor were estimated. Reduced incidence of diseases results in reductions in prevalence and disease-related mortality and morbidity. This leads to improved long term health outcomes and healthcare cost-savings.(18-22) Cohort-based modelling allowed health-related quality of life (HRQoL) and obesity-related health benefits, which are not present in early childhood, to be estimated assuming lingering BMI effects. For weight loss maintained into adulthood, population impact fractions (PIFs) were estimated (30) and used to estimate the consequences of a change in BMI on the incidence of nine obesity-related diseases (ischaemic heart disease, hypertensive heart disease, ischaemic stroke, diabetes, colorectal cancer, kidney cancer, breast cancer, endometrial cancer and osteoarthritis). PIFs were also used to estimate the consequences of a change in MET minutes on physical activity-related diseases (ischemic heart disease, stroke, type 2 diabetes, breast cancer and colon cancer)(31), and published adjustment factors were applied to avoid double-counting.(32)  The model used data from the Australian Health Survey 2011-12 and disease epidemiology from the Global Burden of Disease study (Table 1).(33,34) The change in risk was compared against the counterfactual, where the distributions in the 2010 Australian reference population remained unchanged. Results were presented as life years (LYs) gained, health adjusted life years (HALYs) gained, and healthcare cost-savings from diseases averted. HALYs were estimated by aggregating the population level changes to mortality and morbidity for each disease (using Global Burden of Disease disability weights (36) and the negative HRQoL impacts attributable to BMI in childhood)(37). Incremental cost-effectiveness ratios (ICERs) were calculated and presented on a cost-effectiveness plane. Cost-effectiveness was determined using the threshold of AUD50,000 per HALY gained.(35) All modelling was undertaken in Microsoft Excel 2016. The model is available upon request to the corresponding author. |
| 9 | Page 9, I'm not sure why old data is being used as the reference year (2010), have risk behaviours and prevalence not changed? | The reference year of 2010 was selected for two reasons. Firstly, the Transform-Us! cluster randomised controlled trial (cRCT) was conducted from 2010 to 2012 and so this reference year aligns with both the availability of the epidemiological data to inform the model, and the time period of the cRCT. Secondly, by utilising the ACE-Obesity policy model and the consistent approaches to determining cost-effectiveness adopted within the priority-setting study, the findings from this economic evaluation are comparable with the broader priority-setting study findings.(1) The ACE-Obesity Policy study adopted a reference year of 2010.(1)  Data from the ABS National/Australian Health Survey series indicate no real change in the prevalence of overweight and obesity among children aged 5–14 between 2007–08 and 2017–18.(2) | No changes made (additional to those already made in #8) |
| 10 | Table 1 - aren't the short-term changes (MET and BMI) key modelling variables too? linking to the RR of chronic disease. As per my earlier comment, I see these as key to the CEA findings and would expect these in the main inputs table. The values and ranges and distribution values should also be in Table 1 as per CHEERs. I feel like this data is hidden and not even presented in the Supplementary files. | Thank you for this comment. This data was initially presented in the Results section, but as per comment #7 has now been moved to the Methods section. We have also included the values in Table 1, based on your suggestion. | Addition of intervention effectiveness estimates and data source into Table 1. See paper for details. |
| 11 | Is a 'pert' distribution a triangular distribution? I've not heard this term before. | A pert distribution is defined by the minimum, most likely and maximum values that a variable can take. The mean of the pert distribution is defined as the weighted average of the three parameters, with four times the weight applied to the most likely value. Pert distributions have been applied in other modelled economic evaluations of obesity prevention interventions (for instance, see the studies included in (3)).  The Pert distribution is an alternative to a triangular distribution. The triangular distribution takes the minimum, most likely and maximum parameters but has a smoother shape (i.e. places equal emphasis on the extreme values as the most likely value).  We have added a table note on the Pert distribution to Table 1. | Table note added to Table 1:  * The Pert distribution is defined by the minimum, most likely and maximum values that a variable can take. The mean of the Pert distribution is the weighted average of the parameters (with four times the weight applied to the most likely value). |
| 12 | Results, Page 11, I'm not clear why the sentence on completion rates of the diary use and info on intervention activities is necessary for the cost section. If the quality of the data is being commented on, this would be is the Discussion. | Following guidance from the CHEERS 2022 checklist, the presentation of this information in Additional Files 2 and 3 reports analytic inputs to the economic evaluation. The varied teacher diary response rates between intervention groups are already discussed as a study limitation. | No changes made |
| 13 | Line 248 - comment on good value for money should be left to the Discussion. | We have deleted this from the Results section. | Results, line 293-294::  This, however, suggests some economies of scale in delivery of the combined PA+SB-I intervention.~~, and so while the intervention may have been more costly it may still represent good value for money~~. |
| 14 | Page 11 - effectiveness results are not for this economic evaluation, they already occurred in the trial? So I don't believe they belong here. The HALYs or other effects in the ICERs should be presented here. | We agree and thank the reviewer for this comment. | See changes made in response to #7 |
| 15 | Table 2 - negative ICERs are not recommended to present - just the dominant or dominated result. | We acknowledge that the interpretation of negative ICERs can be challenging, however have presented results here in line with those presented within the wider priority-setting study.(3) While negative values are presented for the total net cost, and net cost per HALY saved, we have also presented the interpretation of these results clearly (i.e. Overall result – Dominant) and table notes to aid in the interpretation of negative values. | No changes made |
| 16 | Table 3 - can the population size of children be added here or in the text so the extrapolation results can be clear. | We have added this detail into the text. | Results, line 329:  Extrapolating the PA-I and SB-I intervention groups to the Australian population of Year 3 children in Australian Government schools (n=184,547 children) led to more significant health benefits and healthcare cost-savings, assuming maintenance of effect over the lifetime (Table 3). |
| 17 | The sustainability of the intervention to keep persons active into adulthood is the big uncertainty where assumptions need to be made over a long period. Is there any evidence among adults who regularly exercise now that they were active as children (self-report or other) to support and strengthen the assumption? | The determinants of physical activity maintenance are complex, and there is mixed evidence on the tracking of physical activity from childhood to adolescence and adulthood (for example, (4-7)). We have added text to this effect to the discussion section.  The assumptions around the maintenance of intervention effect are clearly included in the study limitations. In addition, the effect decay assumptions have been tested in sensitivity analyses (maintenance for 10 years, in line with the findings of a recent review of similar studies).(4) This aligns the methods used in this paper with other similar published economic evaluation studies  We have also noted in the paper:  Limited evidence exists on the long-term maintenance of the intervention effect, although the use of effect estimates at 30 months (i.e. after the 12 month tapered maintenance period) may suggest sustained changes to behaviour amongst intervention participants. | Discussion, line 408-411:  It should, however, be noted that the determinants of physical activity maintenance are complex, and there is mixed evidence on the tracking of physical activity from childhood to adolescence and adulthood (for example, (48,50-52)). This is a significant area for future research. |
| Reviewer #2 | | Author’s response | Changes to document |
| The manuscript aimed to assess the cost-effectiveness of a school-based intervention program targeting increased physical activity (PA) and/or reduced sedentary time among 8-9-year-old children in Australian Government primary schools, utilizing the simulation modeling approach with the validated ACE-Obesity Policy model. While acknowledging the merits of the study, several suggestions are proposed for manuscript improvement. Below are section-specific comments. | | | |
| 18 | Abstract-(Methods): The authors are encouraged to provide additional details on the modeled cost-effectiveness analysis (CEA), including the time horizon, perspectives, measured effects, and sensitivity analyses. | We thank the reviewer for this comment. We have added this detail into the abstract, noting that further inclusion of detail is word limited as per journal guidelines. | Abstract, Methods:  A validated multiple-cohort lifetable model (ACE-Obesity Policy model) estimated the obesity and physical activity-related health outcomes (measured as change in body mass index and change in metabolic equivalent task minutes respectively) and healthcare cost-savings over the cohort’s lifetime from the public-payer perspective, assuming the intervention was delivered to all 8-9 year old children attending Australian Government primary schools. Sensitivity analyses tested the impact on cost-effectiveness of varying key input parameters, including maintenance of intervention effect assumptions.  Abstract, Results:  The PA-I and SB-I interventions remained cost-effective in sensitivity analysis, assuming the full decay of intervention effect after 10 years. |
| 19 | Abstract- (Results): Clarify the wording in the first sentence of the Results section, as it may suggest two separate CE analyses. Specify whether the modeled CE analyses were conducted solely among the intervention arm with a statistically significant effect. | These are separate analyses – comparing either the PA-I or SB-I interventions arms with the comparator. In total, the paper presents the results of four cost-effectiveness analyses (within-trial PA-I arm, within-trial SB-I arm; modelled PA-I arm, modelled SB-I arm). As the paper states in the Results section (lines 292-294), the cost-effectiveness of the PA+SB-I arm was not estimated, given that intervention effects on BMI and sedentary time were not statistically significant in this intervention arm. | No changes made |
| 20 | Abstract- (Results): Present a comparative cost-effectiveness analysis between PA-I and SB-I | The cost-effectiveness analyses undertaken compare the relevant intervention arm (either PA-I or SB-I) to a ‘no intervention’ comparator. This is the most policy and practice relevant comparator (rather than comparing PA-I with SB-I). In addition, the ACE Obesity Policy model compares the change in risk against the counterfactual, where the distributions in the 2010 Australian reference population remained unchanged (as noted in Methods, line 230-232). A comparative cost-effectiveness analysis between the PA-I and SB-I arms is beyond the scope of this economic evaluation as adjusting the counterfactual within the complex and large ACE model would be extremely resource intensive. As previously mentioned, it is most policy and practice relevant to compare the interventions (PA-I, SB-I) with usual practice (i.e. no intervention). | No changes made |
| 21 | Introduction- (Line 85): Elaborate on the mixed results of systematic reviews regarding the effectiveness of school-based interventions. | We have added to this text. | Introduction, line 91-94:  While the effectiveness of school-based interventions has been examined in several systematic reviews, the results have been mixed.(6,8,9) Studies suggest that a large degree of heterogeneity exists in the frequency, duration and content of school-based interventions, and that more focus is needed on understanding both the equity effects and the implementation processes of these complex interventions.(6,8,9) |
| 22 | Introduction- (Lines 89-90): Provide more details on the findings of the systematic review of 23 studies focusing on the economic evaluation of school-based interventions. Justify the need for the current study. | See response to comment #5 | See response to comment #5 |
| 23 | Introduction- (Lines 91-95): Consider moving the paragraph before the objective to the Methods section. | We thank the reviewer for this comment, but feel the flow of the paper is improved if the paragraph remains in its current position. By briefly introducing the Transform-Us! intervention at this stage of the paper, we feel that the reader can gain a better understanding of the context for the economic evaluation. | No changes made |
| 24 | Methods- (Lines 102-103): Clarify the meaning of "costs and outcomes were estimated by a comparison of the intervention and control arms, using intention-to-treat principles within the trial sample (n=1,606 children)." Specify if this is a cost-effectiveness measure (e.g., ICER) or the derivation of cost and effect outcomes from the cluster RCT. | We have added some text to further clarify this point in the introductory paragraph of the Methods section. More detail on these points is provided later in the Methods section. | Methods, line 117-119:  Costs and outcomes were estimated by a comparison of the intervention and control arms, using intention-to-treat principles within the trial sample (n=1,606 children). Modelled cost-effectiveness analysis estimated incremental cost-effectiveness ratios, incorporating lifetime health and cost outcomes attributable to the intervention within the trial sample. |
| 25 | Methods- (Lines 104-105): Define the Australian population of Year 3 children for international readers. | Thank you for this comment, we have further defined this population. | Methods, line 121:  Trial costs and outcomes were then extrapolated to the Australian population of Year 3 students in Government schools (approximately 69% of all fulltime Year 3 children in 2010 (corresponding with the first year of the cRCT), n= 184,547 children aged approximately 8-9 years old),(17) to estimate the potential lifetime health and cost outcomes attributable to the intervention should it be delivered comprehensively throughout Australia. |
| 26 | Methods- (Lines 115-116): Clarify the randomization unit of the parent trial in the Transform-US! study. As Authors mentioned the Transform-US! is a cluster RCT, and also that "cohort of 1,606 children in Year 3 (mean age 8.3 years) at baseline were randomised by school to one of four groups." | Thank you for this comment. We have clarified the wording, and added further information on the randomisation procedure for the reader. | Methods, line 131:  In summary, ~~an cohort of~~ 1,606 children in Year 3 (mean age 8.3 years) at baseline were randomised by school to one of four groups: SB-I targeting reductions in sedentary behaviours; PA-I targeting increases in physical activity; PA+SB-I combining PA-I and SB-I strategies; or, usual curriculum control (C). Schools in low (n=74), mid (n=74) and high (n=71) socioeconomic status areas were randomly ordered with probabilistic weighting according to enrolment number, and invited to participate. Eight schools from low socioeconomic status areas, 11 schools from mid socioeconomic status areas and 1 school from high socioeconomic status area agreed to participate. For randomization, schools in mid and high socioeconomic status areas were combined and schools within each of the two strata were randomly allocated using computer-generated blocks of four by a statistician not involved in the trial.(11) |
| 27 | Methods- (Line 126): Correct the typo 9ie.e., this issue) in the citation (Salmon et al. this issue. (11)). | Apologies for this typo, this has now been amended. | Methods, line 149:  The intervention has been described elsewhere in detail ~~(Salmon et al. this issue .~~.(14) |
| 28 | Methods- (Perspective, time horizon, discount rate): Present two reference case analyses, one from a healthcare-sector perspective and another from a societal perspective, according to the best practices in conducting and reporting cost-effectiveness analyses, | The perspective is clearly stated in Methods, line 151. The perspective utilised is the public payer perspective. This perspective captures the costs and benefits relevant to both health and education in Australia, where universal systems exist. This perspective was also chosen due to the challenges in accurately capturing and including a societal perspective. For example, the Transform-Us! cRCT did not collect data on productivity impacts (e.g. absenteeism, presenteeism) of the intervention. We also did not have data from the cRCT on any impacts in family members, and so these were excluded from the analysis. We have added text to the limitations section in the Discussion, to this effect. While a societal perspective is recommended, a recent systematic review reports that only 35% of included studies adopted a societal perspective, and most of those perspectives did not incorporate all of the possible costs and consequences broadly to society given the challenges in doing so accurately (e.g. in incorporating all downstream costs etc).(8) The Second Panel on Cost-Effectiveness in Health and Medicine (9) notes that analysts may include narrower perspectives than the societal perspective, in order to address specific decision contexts. Given this, we have amended the text in the Methods section to reflect that the recommendations of the Second Panel on Cost-Effectiveness guided the analysis where practicable. | Methods, line 114:  The recommendations of the Second Panel on Cost-Effectiveness and the Consolidated Health Economic Evaluation Reporting Standards (Additional File 1) guided the analysis where practicable.(15,16)  Methods, line 165-168:  The analysis was undertaken from the public-payer perspective. This perspective was identified as most relevant to capture the costs and benefits to both the health and education sectors, given that within the Australian setting universal health and education systems exist.  Discussion, line 417-419:  We were limited in undertaking a societal perspective as per health economics guidance,(10) as data to inform the broader costs and benefits of the intervention (for example, productivity effects) were not available. |
| 29 | Methods- (Measurement of costs): Clearly mention cost categories incorporated in the CEA before elaborating on individual measurements. Include downstream costs (e.g., healthcare utilization) considering the lifetime horizon. | Intervention cost categories are clearly defined in Methods, line 159-163:  The major categories of resource use identified through pathway analysis were: (i) teacher time to prepare intervention delivery; (ii) implementation costs, including equipment provided to schools; and (iii) ongoing costs required if the program was rolled out in the future (e.g., production/distribution costs of newsletters). Costs associated with intervention design and development and the time contributions of children were excluded.  We have clarified the title of this section of the paper.  Downstream costs of healthcare cost-savings from diseases averted, and the methods for estimating them, are described in Methods, line 207-232; also in Table 1.  The time horizon is clearly stated in Methods, line 155-156. | Amended title, Methods, line 174:  *Measurement of intervention costs* |
| 30 | Methods- (Line 154): As a societal perspective is recommended by the best practice in conducting CEA, the team contribution from children or family members may not be excluded from the analyses. | See response to comment #28 | See changes made in response to comment #28 |
| 31 | Methods- (Lines 165-166): Explain why teacher time delivering the intervention was excluded, considering the recommendation to include opportunity costs. | Teacher time to deliver the intervention was excluded based on opportunity cost principles. That is, it was deemed that there was no opportunity cost of delivering the intervention (as teachers would have otherwise spent time delivering similar or other curriculum or educational content to children). We have clarified this in the text. | Methods, line 191-192:  Teacher time to deliver the intervention activity was not included, according to opportunity cost principles, as teachers would otherwise have been required to spend this time delivering similar or other curriculum content. |
| 32 | Methods- (Lines 177-178): Specify whether the assessment was conducted in the current study and provide details of the statistical analyses used. | The paper states:  Measures of intervention effectiveness were based on detailed published study results.(14) Intervention effects (comparison between each experimental group and the control group) related to sedentary time and BMI at 30 months were assessed for statistical significance (p-value <0.05).  To further clarify that effectiveness results were taken from the published study results paper, we have added the reference to the paper to this sentence. | Methods, line 205:  Measures of intervention effectiveness were based on detailed published study results.(14) Intervention effects (comparison between each experimental group and the control group) related to sedentary time and BMI at 30 months were assessed for statistical significance (p-value <0.05).(14) |
| 33 | Methods- (Lines 181-182): Clarify the role of the reduction of BMI z-score in the modeled CEA and how it impacts outcomes. | The methods section detailing the ACE Obesity Policy model provides this detail (page 10, line 210-235). We have added more detail to the modelled CEA approach in comment #8. References to published papers on the modelled approach are also given. | No change made (in addition to those made in #8) |
| 34 | Methods- (Lines 184-185): Provide more details, including an equation, on how changes in sedentary time are converted to changes in MET and their impact on the modeled outcomes. | We have added this detail to the Methods section text. The Methods section already includes information on the impact on modelled outcomes:  Methods, line 229-232.  PIFs were also used to estimate the consequences of a change in MET minutes on physical activity-related diseases (ischemic heart disease, stroke, type 2 diabetes, breast cancer and colon cancer)(11), and published adjustment factors were applied to avoid double-counting.(12)  In addition, references are provided to more publications on the ACE-Obesity Policy model. | Methods, line 222-224:  Statistically significant reductions in sedentary time were converted to a change in metabolic equivalent task (MET) minutes per week.(23) It was assumed that the intervention resulted in the net difference in MET minutes between time spent sitting talking (MET value 1.4) and time spent standing talking (MET value 1.8).(23) This assumption may underestimate results if the reduction in sedentary time equated to a change from sitting to an activity with a higher MET value (e.g., walking-light effort (MET value 2.9)(23)). Statistically significant reductions in sedentary time were multiplied by the difference in MET values (0.4MET), to estimate the change in MET minutes arising from intervention. |
| 35 | Methods- (Lines 191-201): Introduce the ACE-Obesity Policy model at the beginning of the Methods section and provide a brief summary. | We have added this detail to the beginning of the Methods section. | See changes made in response to comment #8  Methods, line 123-124:  Modelled cost-effectiveness was estimated using the well-validated ACE-Obesity Policy model.(18-22,29) |
| 36 | Methods- (Line 206): Specify how HALYs were measured. | We have added text to the methods section detailing the ACE Obesity Policy model and the estimation of HALYs. References to published papers on the modelled approach are also given. | Methods, line 233-236:  The intervention-related changes to the distribution of risk factor/s (BMI, physical activity) on the incidence of disease related to that risk factor were estimated. Reduced incidence of diseases results in reductions in prevalence and disease-related mortality and morbidity. This leads to improved long term health outcomes and healthcare cost-savings.(18-22,29)  Methods, line 252-256:  HALYs were estimated by aggregating the population level changes to mortality and morbidity for each disease (using Global Burden of Disease disability weights (35) and the negative HRQoL impacts attributable to BMI in childhood)(36). |
| 37 | Methods- (Uncertainty and sensitivity analysis): Expand one-way sensitivity analysis to include all key parameters affecting CEA results. | Sensitivity analyses were undertaken on key parameters. Specifically, sensitivity analysis tested the impact on cost-effectiveness results when all teacher preparation time data; and when the assumptions re intervention effect maintenance were varied (to ten years maintenance of effect, in accordance with similar studies from the literature).(4) Given the resources available to conduct this study, these sensitivity analyses were selected as key input parameters that may affect CEA results. We have previously amended the text re the recommendations of the Second Panel on Cost-Effectiveness guiding the analysis where practicable. The selection of key input parameters for testing in sensitivity analysis also corresponds to the methods used in published literature (for example, (3)). | No changes (in addition to those made in response to comment #28) |
| 38 | Methods- (Lines 234-235): Clarify whether the assumption of intervention effect decay after 10 years also applies to both base case and probabilistic sensitivity analyses. | We have added this detail to the Methods section. | Methods, line 246-247  The base case analysis assumed maintenance of intervention effect over the lifetime.  Added text to Table 1:  Base case analysis assumes maintenance of effect over the lifetime. |
| 39 | Methods- Summarize all assumptions made in the simulation modeling CEA. | The methods section summarises key assumptions made in undertaking the modelled economic evaluation. For instance, text on the maintenance of effect, the use of published adjustment factors to avoid double-counting, and the assumptions used to estimate the cost of intervention. In addition, references are provided to publications that comprehensively detail the ACE-Obesity Policy model (including all assumptions, data sources, steps in development etc). | No changes made in addition to those already made in addressing all previous comments. |
| 40 | Methods- Provide cost data for the disease averted resulting in healthcare cost savings | References are provided to publications that comprehensively detail the ACE-Obesity Policy model (including all assumptions, data sources, steps in development etc). Table 1 lists the data source used, and further detail on the methodology is available in the more comprehensive model publications.(1, 13) | No changes made in addition to those already made in addressing all previous comments. |
| 41 | Methods- Clarify the difference between the simulation model of PA-I arm and the SB-I model. Were both the effect of BMI and sedentary time reduction applied in both arms? | The SB-I arm incorporated the effects on BMI and sedentary time. The PA-I arm incorporated effects on BMI only. The detail of this is included in the paper (line 200-207). We have added further clarification on the intervention effect by arm to Table 1. | See Table 1 in the paper, now including details on intervention effect by arm. |
| 42 | Methods- Authors may add a comparative cost-effectiveness analysis between PA-I and SB-I. | See response to comment #20 | No changes made |
| 43 | Results- (Cost of intervention and Effectiveness). Specify how the cost and statistical analyses of the intervention program were conducted in the Methods section and consider adding this to the study objectives or title. | See response to comment #29 | See response to comment #29 |
| 44 | Results- Present the value of key parameters used in the simulation model in Table 1 for replicability. | We have added these in response to comment #10. | See Table 1 in the paper |
| 45 | Results- (Lines 266-267): Mention in the Methods section that the modeled CEA results are also based on the trial population. | The Methods section of the paper now clearly states that the modelled CEA results are based on the findings from the trial, and reported in Salmon et al. (14) | See response to comment #7 |
| 46 | Results- (Table 2): Clarify the time horizon for the modeled trial population and the modeled decrease in BMI z-score. | We have clarified this within the text. | Table 2 and Table 3 headings amended:  Table 2- Modelled trial population cost-effectiveness results, over the lifetime  Table 3 – Cost-effectiveness results, extrapolating PA-I and SB-I to the Australian population of Year 3 children in Government schools, over the lifetime |
| 47 | Results- (Line 296): Clearly indicate that Figure 1 represents the results of probabilistic sensitivity analysis. | We have added text to the figure notes, so this is very clear. | Figure 1, Figure notes:  *Figure notes:* AUD= Australian dollars. Parameter uncertainty is demonstrated through probabilistic sensitivity analysis. |
| 48 | Results- (Figure 2): Explain why incremental health benefits (HALYs) drop near zero around 2030. | This is explained in the Results, line 352-359:  Figure 2 demonstrates the intervention costs, cost offsets and health gains over the lifetime of the cohort for the SB-I and PA-I intervention groups. Whilst the costs of the intervention are incurred upfront, the benefits of the intervention in terms of healthcare cost-offsets of diseases averted do not start to accrue until at least 20 years in the future and peak at around the time that the cohort of Year 3 children are 70 years of age. Health benefits related to an improvement in health-related quality of life in childhood occur in the short-term, long before the health benefits related to the prevention of chronic diseases occur (Figure 2). | No changes made |
| 49 | Discussion- (Lines 343-344): Acknowledge that most implementation costs (activities associated with study reach and adoption in the school setting and training costs) were not captured, and caution against prematurely declaring Transform-US as low cost. | We thank the reviewer for this comment, and have further added text to this effect in the limitations. | Discussion, line 421-424:  In addition, the costs of scaling up the intervention to delivery across all government schools were estimated and therefore may not be reflective of actual implementation costs. This is an area for more exploration; in particular, the impacts of implementation at scale on overall cost-effectiveness warrant further research. A significant area for future work is a comprehensive assessment of how to better incorporate considerations of scale into health economic analysis, particularly important for building the evidence of cost-effectiveness for preventive public health interventions at the population level. While our analyses did not include costs associated with intervention reach and adoption in the school setting, it could be hypothesized that incorporation of the intervention into the Australian curriculum could help to minimize such costs. |
| 50 | Discussion- Compare findings with other similar studies in the discussion. | Findings are compared with similar studies in the Discussion, line 395-403:  While there are methodological differences between previous studies and this trial that make a direct comparison problematic, the results demonstrate the potential value for money of the *Transform-Us!* physical activity and sedentary behaviour interventions, when compared to other obesity prevention interventions that have been undertaken in Australian school or community settings (Additional File 5). When compared to interventions estimated in an Australian priority-setting study that did use consistent methodologies and where comparability between the results from this study are valid, the *Transform-Us!* PA and SB interventions ranked as the seventh and eighth most cost-effective interventions.(22) | No additional changes made |
| 51 | Discussion- (Lines 358-359): Micro-costing of intervention delivery is a strength, but it is jeopardized by the lack of information on methods. | In response to previous comments, we have strengthened the Methods section of the paper. We thank the reviewers for their comments. | No additional changes made |

REFERENCES

1. Ananthapavan J, Sacks G, Brown V, Moodie M, Nguyen P, Barendregt J, et al. Priority-setting for Obesity Prevention - the Assessing Cost-Effectiveness of Obesity Prevention Policies in Australia (ACE-Obesity Policy) study. PLOS One. 2020;15: e0234804.

2. Australian Bureau of Statistics. Australia's children Canberra: ABS; 2022 [Available from: <https://www.aihw.gov.au/reports/children-youth/australias-children/contents/health/overweight-obesity>.

3. Ananthapavan J, Sacks G, Brown V, Moodie M, Nguyen P, Veerman L, et al. Priority-setting for obesity prevention-The Assessing Cost-Effectiveness of obesity prevention policies in Australia (ACE-Obesity Policy) study. PLoS One. 2020;15(6):e0234804.

4. Brown V, Ananthapavan J, Sonntag D, Tan EJ, Hayes A, Moodie M. The potential for long-term cost-effectiveness of obesity prevention interventions in the early years of life. Pediatric Obesity. 2019;0(0):e12517.

5. Herman KM, Craig CL, Gauvin L, Katzmarzyk PT. Tracking of obesity and physical activity from childhood to adulthood: The Physical Activity Longitudinal Study. International Journal of Pediatric Obesity. 2009;4(4):281-8.

6. Gordon-Larsen P, Nelson MC, Popkin BM. Longitudinal physical activity and sedentary behavior trends: Adolescence to adulthood. American Journal of Preventive Medicine. 2004;27(4):277-83.

7. Batista MB, Romanzini CLP, Barbosa CCL, Blasquez Shigaki G, Romanzini M, Ronque ERV. Participation in sports in childhood and adolescence and physical activity in adulthood: A systematic review. Journal of Sports Sciences. 2019;37(19):2253-62.

8. Onyimadu O, Violato M, Astbury NM, Hüls H, Heath L, Shipley A, et al. A systematic review of economic evaluations of interventions targeting childhood overweight and obesity. Obesity Reviews. 2023:e13597.

9. Sanders GD, Neumann PJ, Basu A, Brock DW, Feeny D, Krahn M, et al. Recommendations for Conduct, Methodological Practices, and Reporting of Cost-effectiveness Analyses: Second Panel on Cost-Effectiveness in Health and Medicine. Jama. 2016;316(10):1093-103.

10. Sanders GD, Neumann PJ, Basu A, Brock DW, Feeny D, Krahn M, et al. Recommendations for conduct, methodological practices, and reporting of cost-effectiveness analyses: second panel on cost-effectiveness in health and medicine. JAMA. 2016;316(10):1093-103.

11. Danaei G, Ding EL, Mozaffarian D, Taylor B, Rehm J, Murray CJ, et al. The preventable causes of death in the United States: comparative risk assessment of dietary, lifestyle, and metabolic risk factors. PLoS Med. 2009;6(4):e1000058.

12. Cobiac L, Vos T, Veerman L. Cost-effectiveness of Weight Watchers and the Lighten Up to a Healthy Lifestyle program. Australian and New Zealand Journal of Public Health. 2010;34(3):240-7.

13. Ananthapavan J, Sacks G, Brown V, Moodie M, Nguyen P, Barendregt J, et al. Assessing Cost-effectiveness of Obesity Prevention Policies in Australia. Melbourne: Deakin University; 2018.

14. Salmon J, Arundell L, Cerin E, Ridgers ND, Hesketh KD, Daly RM, et al. Transform-Us! cluster RCT: 18-month and 30-month effects on children’s physical activity, sedentary time and cardiometabolic risk markers. British Journal of Sports Medicine. 2022:bjsports-2022-105825.
